# Pockels effect of silicate glass-ceramics: Observation of optical modulation in Mach–Zehnder system

**DOI:** 10.1038/srep12176

**Published:** 2015-07-17

**Authors:** Kazuki Yamaoka, Yoshihiro Takahashi, Yoshiki Yamazaki, Nobuaki Terakado, Takamichi Miyazaki, Takumi Fujiwara

**Affiliations:** 1 Department of Applied Physics, Tohoku University, 6-6-05 Aoba, Aoba-ku, Sendai 980-8579, Japan; 2 Department of Instrumental Analysis, Tohoku University, 6-6-11 Aoba, Aoba-ku, Sendai 980-8579, Japan

## Abstract

Silicate glass has been used for long time because of its advantages from material’s viewpoint. In this paper, we report the observation of Pockels effect by Mach–Zehnder interferometer in polycrystalline ceramics made from a ternary silicate glass via crystallization due to heat-treatment, i.e., glass-ceramics. Since the silicate system is employed as the precursor, merits of glass material are fully utilized to fabricate the optical device component, in addition to that of functional crystalline material, leading us to provide an electro-optic device, which is introducible into glass-fiber network.

Basically, there are two important components for optical telecommunications. One is optical-control system based on nonlinear-optical crystal, which is structurally noncentrosymmetric. Particularly, lithium niobate (LiNbO_3_) single crystal has been employed because a notable electro-optic (EO) effect due to the large spontaneous polarization, i.e., Pockels effect, can manage the optical signals^1^,^2^. Nevertheless, single crystal with optical grade is usually fabricated by a massive growth equipment (e.g., Czochralski technique) so that the production cost could be considerably high. Other is signal propagation system based on silica (SiO_2_)-glass fiber^3^,^4^. Reason why the huge amount of fibers could be produced is because the low material of silicate minerals is easily available from the earth crust. In addition, glass has a random structure without translation symmetry, resulting in the optical isotropy and good formability. Furthermore, glass material acquires various properties/functions by addition of network-modifier (e.g., alkali, alkali-earth and rare-earth oxides) and intermediate oxide (e.g., transition-metal oxide). However, the structural isotropy leads the glass material to forbid the macroscopic polarization, meaning that Pockels effect is essentially absent. In these circumstances, if we can made the EO-device from silicate glass, which is operated on the basis of Pockels effect, the issues concerning the optical crystal could be resolved, and new application of glass material to an active optical-device is opened.

In this article, a fundamental procedure to realize an optical device component consisting of a polycrystalline material obtained from glass precursor, i.e., glass-ceramic (GC) processing, is presented. We demonstrated the signal-intensity modulation based on Pockels effect in transparent GCs obtained from silicate glass, in which nonlinear-optical crystal is crystallizable: A silicate mineral, fresnoite (Ba_2_TiSi_2_O_8_)^5^ and its derivatives (Sr_2_TiSi_2_O_8_ and Ba_2_TiGe_2_O_8_)^6^,^7^ have a spontaneous polarization due to the alignment of pyramidal TiO_5_ units along to *c*-axis, resulting an excellent piezoelectric and optical properties^8^,^9^,^10^,^11^,^12^. For example, second-order optical nonlinearity for the Ba_2_TiSi_2_O_8_-, Sr_2_TiSi_2_O_8_- and Ba_2_TiGe_2_O_8_-surface-crystallized GCs, which is measured by means of second-harmonic generation (SHG), has been reported to be *d*_33_ ~ 13 pm/V, *d*_33_ ~ 7.2 pm/V and *d*_33_ ~ 22 pm/V, respectively, and is closely related to the ratio of lattice constant of a-axis to that of lattice constant of *c*-axis, i.e., *a*/*c* (or tetragonality)^12^. Fresnoite-crystallized GCs have been intensively studied from viewpoint of both fundamental glass-science and practical application so far^13^,^14^,^15^,^16^,^17^,^18^,^19^,^20^,^21^,^22^,^23^. In this study, we focused on 35SrO–20TiO_2_–45SiO_2_ (STS45) glass, which singly crystallizes fresnoite-type Sr_2_TiSi_2_O_8_. The STS45 glass possesses the following features:

- “Perfect surface crystallization (PSC)” occurs, in which the single-crystal domains grow from glass surfaces, and eventually their growth fronts impinge on each other. The resulting GCs show a uniform and dense texture of fresnoite-phase with the large thickness (~0.5 mm) and strong orientation to polar *c*-axis^24^,^25^.
- The STS45 glass has the composition that Sr_2_TiSi_2_O_8_ phase is added with excess SiO_2_ to improve the glass formability, leading the glass to be nonstoichiometric. During PSC, the excess component appears as amorphous nanoparticles, which are frozen in the fresnoite single-crystal domain, i.e., nanoparasites^26^.
- The PSC-GC samples realize a high visible transmittance and/or low optical-loss (~0.6–0.8 dB/cm), comparable to that of an optical waveguide in LiNbO_3_ single crystal^27^. This is due to nano-sizing of the SiO_2_ parasites (less than visible wavelength), which minimizes the Rayleigh scattering.

## Results

### Preliminary assessment

**Figure 1:**
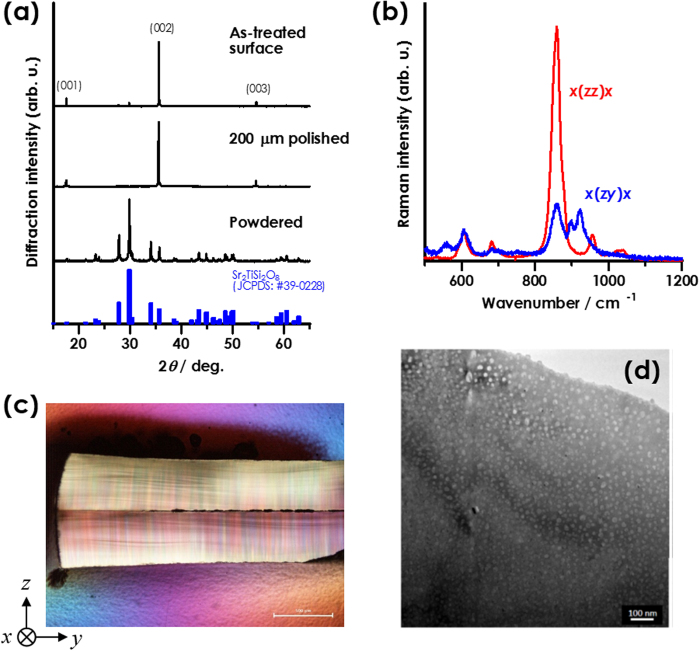
Characterizations of the glass sample subjected to the isothermal heat-treatment: (**a**) XRD patterns for the surface regions of sample and its powdered state. The patterns revealed the a few strong peaks corresponding to (0 0 *n*) planes (*n* = 1, 2, 3), i.e., formation of oriented fresnoite texture to the polar *c*-axis; (**b**) Raman spectra measured by different polarization conditions, i.e., *x*(*zz*)*x* and *x*(*zy*)*x*, in the cross-section of PSC-GCs. [their configurations are shown in (**c**)]; (**c**) polarization microscope image for the cross-section of the PSC-GC sample; (**d**) TEM image in the domain structure.

Firstly, we prepared the PSC-GC sample with Sr_2_TiSi_2_O_8_ phase, and performed different microscopic observations. We could obtain the GCs by means of heat-treatment at 940 °C for 3 h. The XRD measurement on the surface region revealed the orientation to polar *c*-axis for the singly-crystallized Sr_2_TiSi_2_O_8_ phase [Fig. 1(a)], and particularly an interior of the crystalline texture, which is obtained by surface-polishing, indicated the significant orientation In addition, polarization micro-Raman measurement was performed in the cross-section of PSC-GC sample. The Raman observation revealed the difference in spectrum between the polarization conditions [Fig. 1(b)]: In *x*(*zz*)*x* polarization, relatively strong signal due to the Raman mode of Ti‒O* bond along *c*-axis, corresponding to the apex of pyramidal TiO_5_ unit, was observed at ~860 cm^−1^ 28,29 in comparison to the spectrum in *x*(*zy*)*z* polarization. The Raman result is reasonable to the result of XRD, i.e., strong c-orientation of crystallized Fresnoite phase. Formation of crystal nuclei on glass surface (based on inhomogeneous nucleation) and subsequent crystal growth results a highly-oriented crystalline texture, so-called “surface crystallization”. In surface crystallization, direction of evolved nuclei is basically randomly oriented and the orientation starts during the growth process owing to geometric selection^30^. However, Wisniewski *et al.* found that in case of the PSC the crystal nuclei roughly orient at the nucleation stage, i.e., orientation nucleation^31^. The strong orientation of crystallized fresnoite phase and its dense texture are considered to be due to the orientation nucleation. Furthermore, we also observed the domain structure (width: ~10–20 μm) with clear retardation, corresponding to the presence of optically-anisotropic regions and impingement of the growth fronts of fresnoite phase [Fig. 1(c)]. Furthermore, the numerous nanoparticles (size: ca. 10 nm) were observed in the GC sample; in other word, “nanometric parasites” in the crystal domain [Fig. 1(d)]. In the previous studies^25^,^32^, the nanometric parasites are due to the nanometric phase-separation into stoichiometric fresnoite-component and residual SiO_2_ component, which finally transform to the crystal-domain and nanometric parasites, respectively. The assessment indicates that the sample obtained in this study is compatible with the PSC-GCs reported so far^24^‒^26^, and consequently it is considered that the obtained sample acquires the PSC features of i) to iii).

### PFM observation in PSC-GCs

**Figure 2:**
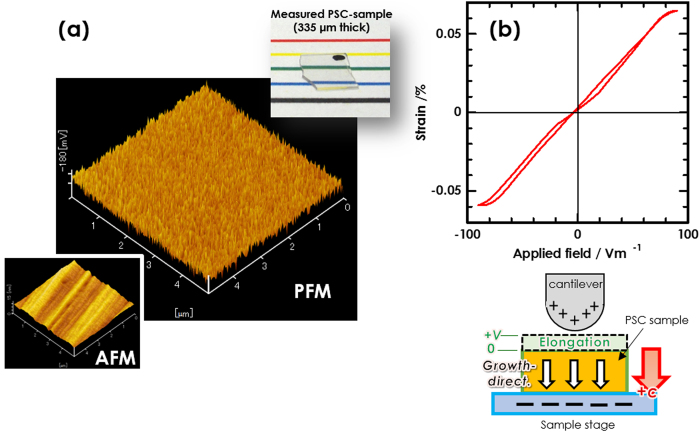
Piezoelectric responce in the PSC-GC sample: (**a**) PFM image of the sample surface together with the corresponding AFM image. The measurement was conducted to the PSC sample, which is subjected to mirror-polishing, with thickness of ca. 0.3 mm (inset); (**b**) strain-electric-field curve for the PSC sample and the schematics of sample settlement. The electric field was applied to the direction of crystal-growth of fresnoite phase.

Prior to EO measurement, we characterized the PSC-GCs by means of PFM because the crystallizing fresnoite-phase is a piezoelectrics originating in the spontaneous polarization. The PFM revealed a uniform distribution of surface potential [Fig. 2(a)], which is measured to be 201 ± 6 mV. A piezoelectric constant, *d*_33_, of fresnoite-type phases is positive^9^,^11^ so that if we apply a positive electric-field along the +*c*-direction of the domains, the values of strain (or displacement) could be positive. As a result, the electric-field-induced strain could be observed in the PSC-GC sample with polar *c*-orientation [Fig. 2(b)]. Since the crystal-growth direction of fresnoite domains in the PSC sample are downward in the measurement system (seen as the schematics), the PFM also revealed that the direction of preferable crystal-growth for fresnoite phase is identical to the +*c*-direction. This is probably significant for the observation of Pockels effect in polycrystalline ceramics. Thus, it was found that the domain texture due to PSC is considerably homogeneous on the basis of piezoelectric point of view.

### Evaluation of Pockels constants in PSC-GCs

Optical system of Mach–Zehnder interferometer is often employed for the evaluation of optical-grade single crystal, but is not suitable for that of polycrystalline material (ceramics) because of much less transparency. However, the PSC-GCs possesses the low optical loss, leading us to expect to perform the quantitative evaluation of EO effect.

**Figure 3:**
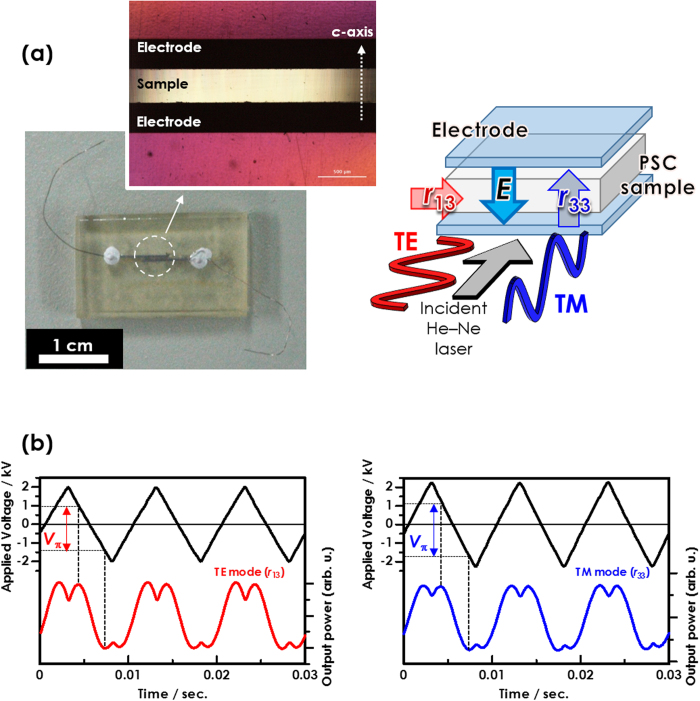
Result of optical modulation observed in the PSC sample by applied voltage (@100 Hz): (**a**) Prepared sample for EO-measurement (appearance and the enlarged picture) and the schematics of polarization condition. The details of preparation and evaluation are described in the part of Methods; (**b**) evaluation of *r*_13_ and *r*_33_. The Pockels constants could be obtained depending on the polarization of incident light. The optical intensity was modulated in accordance with the variation in electric field.

We prepared the sample for EO-observation using the PSC-GCs [Fig. 3(a)], and applied the AC electric field to the sample in Mach–Zehnder system. We could observe the variation in intensity of interference of light as a function of the applied voltage, i.e., the optical modulation based on Pockels effect [Fig. 3(b)], demonstrating that active EO function in polar-oriented ceramics. In addition, we evaluated the Pockels constants to be *r*_13_ ~ 2.7 pm/V and *r*_33_ ~ 2.3 pm/V, which are obtained from the half-wave voltage (*V*_π_) in TE (transverse electric wave) and TM (transverse magnetic wave) polarization, respectively. These values are comparable to that of *r*_22_ of LiNbO_3_. Furthermore, the evaluation by means of Mach–Zehnder system strongly suggests not only the optical modulation function in the PSC-GCs, but also its optical transparency applicable to practical photonic device.

## Discussion

LiNbO_3_ has been utilized as nonlinear optical crystal exclusively for the EO-driven devices. On the other hand, the single crystal materials are much less flexible and workable to obtain special shape/form, resulting the connector loss between the fiber and crystal. In order to use EO device in fiber-based network system, “fiber-form device” is considered to be one of the best way for the easy connection to conventional fiber. Although silica glass-fibers with second-order optical nonlinearity have been fabricated by means of poling technique so far^33^,^34^, the optical nonlinearity could be reduced due to the structural relaxation in the poled glass^35^. This is a fatal problem for the long-term reliability.

Hane *et al.* successfully fabricated the GC-fiber, in which domains of fresnoite phase radially crystallizes, in ternary BaO–TiO_2_–GeO_2_ system glass^36^. Subsequently, Ohara *et al.* created the glass-fiber with double-clad structure, in which the first clad crystallizes the fresnoite-type Ba_2_Ti(Ge,Si)_2_O_8_, and demonstrated optical-attenuator function based on Pockels effect^37^. In the double-clad fiber, not the core but the first clad was crystallized selectively to operate the optical signal because crystallization phenomenon usually provides the scattering center/interface. This was the reason why we hesitated the use of crystallization in core region. Nevertheless, the propagation loss in this EO fiber device is considerably large^37^, which is attributed to inhomogeneous texture of the crystallized fresnoite at the interface between the core and first-clad. Therefore, quantitative evaluation of Pockels effect has not been performed in the GC-fiber devices. Iwafuchi *et al.* measured the Pockels constants in Ba_2_TiSi_2_O_8_-crystallized GCs by the Mach–Zehnder system, i.e., *r*_13_ = 3.15 pm/V, *r*_33_ = 1.00 pm/V, and the polarization dependence on the EO effect, i.e., *r*_13_/*r*_33_, is evaluated to be ~3, which corresponds to the value of Ba_2_TiSi_2_O_8_ single crystal^38^. Meanwhile, the PSC-GCs with Sr_2_TiSi_2_O_8_ phase in this study possesses much less dependence, i.e., *r*_13_/*r*_33_ ~ 1.2, despite that both crystal structure of Sr_2_TiSi_2_O_8_ phase is identical to that of Ba_2_TiSi_2_O_8_ phase in the tetragonal system. Iwafuchi also suggested that a residual stress largely affects the EO constants in the fresnoite glass-ceramics^38^. Since the second-order optical nonlinearity of Sr_2_TiSi_2_O_8_ phase, which is evaluated by SHG measurement, is smaller than that of Ba_2_TiSi_2_O_8_ phase^12^, a possible reason of the difference in *r*_13_/*r*_33_ ratio is supposed to be the increase of *r*_33_ in PSC-GCs due to the internal stress. Although more study is indeed necessary to explain the less dependence on Pockels constants, the result leads us to largely expect that the isotropic optical modulations are operated if different polarization signals are incident on the PSC material with circular symmetric domain structure, i.e., fiber-form PSC-GC device.

In concluding remarks, a sintered ceramics possessing optical transparency and high orientation has been prepared by the combination of a high magnetic field (>10 T) and slip casting technique^39^. On the other hand, the PSC is possible to prepare the material by heat treatment for a short time, and the oriented texture is obtained on the basis of a self-organization. In addition, the glass precursor in multicomponent system enables the advantageous fabrication to mass-production and yield rate. Therefore, the PSC-GCs help to fabricate the practical fiber-form EO-device, which integrates the control and propagation system, and is expected to support the development of telecommunication network. Furthermore, this study also suggests the application of polycrystalline ceramics to sophisticated optical devices as alternative material of nonlinear optical single-crystal.

## Methods

### Sample preparation

The composition of precursor glass was 35SrO−20TiO_2_−45SiO_2_ glass, which indicates the perfect-surface-crystallization (PSC), and was prepared through a conventional melt-quenching technique. The obtained glass was polished to get a mirror surface and then cut into several pieces with dimension of ~10 mm × 10 mm × 1 mm, and subsequently was subjected to isothermal heat-treatment at 940 °C (at 920 °C for sample in polarization Raman study) for 3h in an electric furnace in air in order to fabricate the PSC-GCs. The PSC-GC sample was characterized by means of X-ray diffraction (XRD) analysis, polarization micro-Raman spectroscopy constructed by an Ar^+^-gas laser operating at 514.5 nm and a system consisting of a triple-grating monochromator and liquid-nitrogen-cooled charge-coupled device detector (HORIBA-Jobin Yvon, T64000), transmission electron microscopy (TEM), piezoelectric force microscopy (PFM), and optical interferometer. In the measurement by PFM and interferometer, we used a piece of the plate, which is cut off from the PSC-GCs and is mirror-polished. Particularly, for the evaluation of Pockels constants by interferometer, the piece was sandwiched with the Al-electrodes, which are placed in perpendicular to the polar *c*-axis. The assembly was coated with a photo-curable resin for electrical insulating, and then mirror-polished on the laser-incident and outgoing sides. In order to apply an AC electric field, Ag-conducting wires were put on the electrodes [*cf.* Fig. 3(a)].

### Evaluation of electro-optic constants

In order to evaluate the Pockels constants, the Mach–Zehnder interferometer system was utilized. The detailed setup was referred to the report by Iwafuchi *et al.*^38^: The assembled sample was settled on an arm of the system, and linear polarized He-Ne laser beam (633 nm) was introduced into the sample using an objective lens. The triangle-wave voltage (*V*_pp_ ~ 4 kV; 100 Hz) was applied to the sample and the optical output power was detected to estimate the *V*_π_. In addition, the refractive index was measured with a prism coupling technique.

A general expression concerning the Pockels constant (*r*) and *V*_π_ is defined by following equation:


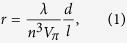


where λ is the wavelength (633 nm in this study), *n* the refractive index (1.76 at 633 nm), *d* the electrode distance (0.29 ± 0.02 mm), and *l* the sample length (4.76 mm). Because of the crystal symmetry in fresnoite, the nonzero tensors of the constants are *r*_13_ (= *r*_23_), *r*_33_, and *r*_42_ (= *r*_51_). In this measurement, the polar *c*-axis is parallel to the applied voltage so that the tensor components of *r*_13_ and *r*_33_ can be evaluated individually by change in the polarization condition of the laser beam, i.e., TE- and TM-mode, respectively. In the *r*_13_- and *r*_33_-evaluation, we used the refractive index for ordinary wave (*n*_o_ = 1.7638 at 633 nm), which is experimentally obtained in the precursor STS45 glass, because fresnoite single crystal possesses a quite small anisotropy in refractive index (*n*_o_ = 1.7613 and *n*_e_ = 1.7592)^10^.

## Additional Information

**How to cite this article**: Yamaoka, K. *et al.* Pockels effect of silicate glass-ceramics: Observation of optical modulation in Mach-Zehnder system. *Sci. Rep.* **5**, 12176; doi: 10.1038/srep12176 (2015).
